# Development of a Synthetic Population Model for Assessing Excess Risk for Cardiovascular Disease Death

**DOI:** 10.1001/jamanetworkopen.2020.15047

**Published:** 2020-09-01

**Authors:** Mary G. Krauland, Robert J. Frankeny, Josh Lewis, LuAnn Brink, Eric G. Hulsey, Mark S. Roberts, Karen A. Hacker

**Affiliations:** 1 Department of Health Policy and Management, University of Pittsburgh Graduate School of Public Health, Pittsburgh, Pennsylvania; 2 Public Health Dynamics Laboratory, University of Pittsburgh Graduate School of Public Health, Pittsburgh, Pennsylvania; 3 Allegheny County Department of Health, Pittsburgh, Pennsylvania

## Abstract

**Importance:**

Evaluating the association of social determinants of health with chronic diseases at the population level requires access to individual-level factors associated with disease, which are rarely available for large populations. Synthetic populations are a possible alternative for this purpose.

**Objective:**

To construct and validate a synthetic population that statistically mimics the characteristics and spatial disease distribution of a real population, using real and synthetic data.

**Design, Setting, and Participants:**

This population-based decision analytical model used data for Allegheny County, Pennsylvania, collected from January 2015 to December 2016, to build a semisynthetic population based on the synthetic population used by the modeling and simulation platform FRED (A Framework for Reconstructing Epidemiological Dynamics). Disease status was assigned to this population using health insurer claims data from the 3 major insurance providers in the county or from the National Health and Nutrition Examination Survey. Biological, social, and other variables were also obtained from the National Health Interview Survey, Allegheny County, and public databases. Data analysis was performed from November 2016 to February 2020.

**Exposures:**

Risk of cardiovascular disease (CVD) death.

**Main Outcomes and Measures:**

Difference between expected and observed CVD death risk. A validated risk equation was used to estimate CVD death risk.

**Results:**

The synthetic population comprised 1 188 112 individuals with demographic characteristics similar to those of the 2010 census population in the same county. In the synthetic population, the mean (SD) age was 40.6 (23.3) years, and 622 997 were female individuals (52.4%). Mean (SD) observed 4-year rate of excess CVD death risk at the census tract level was –40 (523) per 100 000 persons. The correlation of social determinant data with difference between expected and observed CVD death risk indicated that income- and education-based social determinants were associated with risk. Estimating improved social determinants of health and biological factors associated with disease did not entirely remove the excess in CVD death rates. That is, a 20% improvement in the most significant determinants still resulted in 105 census tracts with excess CVD death risk, which represented 24% of the county population.

**Conclusions and Relevance:**

The results of this study suggest that creating a geographically explicit synthetic population from real and synthetic data is feasible and that synthetic populations are useful for modeling disease in large populations and for estimating the outcome of interventions.

## Key Points

**Question:**

Can synthetic populations that statistically mimic real populations in characteristics and spatial distribution of disease be constructed with real and synthetic data, and are these synthetic populations useful in designing and targeting interventions?

**Findings:**

In this decision analytical model of a synthetic population of 1 188 112 individuals with an excess risk for cardiovascular disease death, the modeling process created was validated through identifying the correlation of cardiovascular disease death risk with social and biological risk factors.

**Meaning:**

This study suggests that spatially explicit modeling using a synthetic population is a feasible way to estimate disease risk and the implication of population-level interventions.

## Teaser

This decision analytical model describes the use of a semisynthetic population to identify the distribution of excess cardiovascular death risk and its correlation with social and biological risk factors.

## Introduction

Evaluating the association of social determinants of health with chronic diseases at the population level requires access to individual-level factors of disease, which are rarely available for large populations. Synthetic populations are a possible alternative for this purpose. A synthetic population is based on and representative of a subset of characteristics of a real population but has no personally identifiable information.^1,2,3,4,5,6,7,8,9^ A Framework for Reconstructing Epidemiological Dynamics (FRED) is an agent-based modeling platform that uses a synthetic population created from the US census data, land use data, and school and workplace data.^4,7^ This population is statistically congruent with the census tract–level population in household demographic characteristics, household location, and income.

Cardiovascular disease (CVD) is one of the leading causes of mortality in the United States, with estimates of CVD death exceeding 600 000 every year.^10,11^ Evidence has shown that social determinants of health (socioeconomic position, social support, access to care, and residential environment) are associated with CVD, but the direct or indirect role that these social determinants play in CVD is not clear.^12,13,14,15,16,17,18,19,20,21,22,23^

Allegheny County is a southwest county in the state of Pennsylvania with a population of approximately 1.2 million and a large metropolitan area as well as surrounding suburbs. This county has a higher-than-average prevalence of CVD than other counties in Pennsylvania. As part of a project to improve health through multisector data-sharing collaborations, the Allegheny County Health Department (ACHD) collected census tract–level public health, human services, economic development, health insurance claims, and transportation data sets. These datasets were obtained from Allegheny County data, the US Census Bureau, other government agencies, and health insurance providers.

In this study, we used these data sets and the FRED synthetic population to create a census tract–specific, geospatially accurate model of excess CVD death risk and associated risk factors. To validate the synthetic population and to demonstrate how such a population can be used, we explored the correlation of social determinants of health with CVD death risk and estimated the potential association of interventions with reduced CVD death risk. This combination of population-level and simulated data represents a novel creation of a statistically realistic synthetic population with demographically reasonable values for variables associated with CVD. We investigated whether such populations could be useful in identifying target areas for interventions and testing the local implications of geospatially targeted interventions.

## Methods

This decision analytical model was approved by the University of Pittsburgh Institutional Review Board. Informed consent was not needed because the study used data from public sources and contained no identifiable information. We followed the non-cost-related Consolidated Health Economic Evaluation Reporting Standards (CHEERS) reporting guideline.

### Synthetic Population

**Figure 1.**
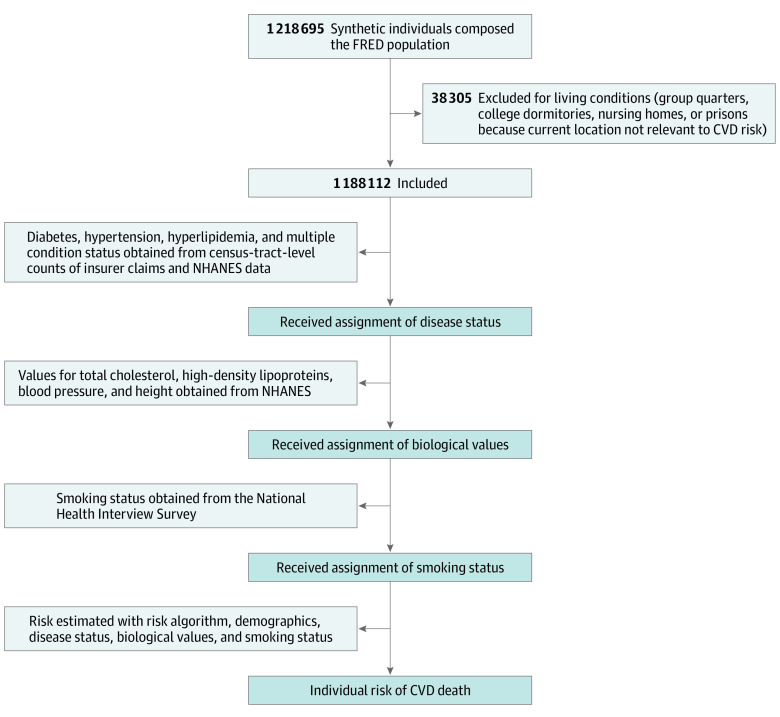
Flowchart for Population Creation and Estimation of Cardiovascular Disease (CVD) Deaths FRED indicates A Framework for Reconstructing Epidemiological Dynamics; NHANES, National Health and Nutrition Examination Survey.

We built a semisynthetic population with demographic characteristics and disease characteristics based on the synthetic population used in the FRED modeling and simulation platform (Figure 1).^4,7^ Disease claim counts obtained from the 3 major health insurers in Allegheny County were used to assign relevant disease conditions to the FRED synthetic population on a census tract basis. For the population outside of the claims data, we obtained disease status from the National Health and Nutrition Examination Survey (NHANES).^24^ Other required variables were obtained from the NHANES and the National Health Interview Survey.^25^ A detailed description of the population creation process is provided in the eMethods in the Supplement. Data were collected from January 2015 to December 2016.

Data on CVD deaths were obtained from the death certificates in Allegheny County. Age-adjusted rates of CVD deaths from January 1, 2010, to December 31, 2013, were calculated and applied to the 2010 census population by census tract (rate × population).

A 5-year risk of CVD death was assigned to each individual in the synthetic population, using a published risk equation.^26^ Risk was scaled to 4 years to match death certificate data and was summed over each census tract. Zero risk was assigned to individuals aged 18 years or younger. To evaluate the reliability of the risk equation used for estimating CVD death rate risk, we performed a linear regression of observed from expected CVD death risk (eAppendix 1 and eFigures 1-3 in the Supplement).^27^ The CVD death risk difference was calculated by subtracting the observed 4-year risk per census tract from the expected 4-year risk per census tract and then was normalized to a risk per 100 000 persons.

Social determinants of health data for the Data Across Sectors for Health project were collected by the ACHD and partners of the Allegheny Data Sharing Alliance for Health (eAppendix 2 and eTable 1 in the Supplement). These free data are hosted by the Western Pennsylvania Regional Data Center at the University of Pittsburgh Center for Social and Urban Research and maintained and updated by the ACHD.

### Statistical Analysis

We performed spatial clustering analysis using GeoDa, version 1.12 (eAppendix 3 in the Supplement)^28^ and all statistical analyses using R, version 3.1.0 (R Foundation for Statistical Computing).^29^ Simple and multivariate linear regression models were used to evaluate the association of single and multiple social determinants with excess CVD death risk. A 2-tailed Pearson correlation coefficient was calculated to ascertain the correlation among social determinants. A value of *P* < .0025 was considered statistically significant for univariate regression, using Bonferroni correction to account for multiple comparisons.

Multivariate models were created first from income-based variables, and stepwise variable elimination was performed to improve the model fit. High school education was added to the best income model. The effects of diabetes, hypertension, and hyperlipidemia were also modeled by multivariate regression. Both the income and education model and the combined social and biological model were used to estimate the effects of changing levels of associated variables. We tested the potential outcome of improving social determinants by modifying the determinant value by census tract. When possible, we increased values (percentage of high school graduates and median income) or decreased values (percentage of households receiving food stamps, percentage without jobs, percentage with diabetes, percentage with hypertension, and percentage with hyperlipidemia) by 10% or 20%. For the percentage of high school graduates, values of 90% or higher were increased to 100%.

Census tracts were ranked for the level of each social determinant, and mean ranks were calculated for each tract to get an overall ranking. Data analysis was performed from November 2016 to February 2020.

## Results

The synthetic population consisted of 1 188 112 individuals and was similar in demographic characteristics to the 2010 census (real) population at the county level but varied by census tract (Table 1). In the synthetic population, the mean (SD) age was 40.6 (23.3) years, and 622 997 were female (52.4%) and 565 115 were male (47.6%) individuals. Census tract populations varied widely in size and in demographic characteristics, disease status, and personal characteristics, including blood pressure, total cholesterol, and high-density lipoprotein cholesterol. However, most mean values clustered around local, state, or national population means (Table 1).

**Table 1.** Characteristics of Synthetic Population

Characteristic	Population values, No. (%)^**a**^	
	Synthetic population	2010 Census population^**b**^
Total population, No.	1 188 112^c^^,^^d^	1 223 348
Age range, y		
0-4	63 016 (5.3)	63 614 (5.2)
5-9	67 435 (5.7)	61 167 (5.0)
10-14	71 306 (6.0)	70 954 (5.8)
15-19	72 376 (6.1)	79 518 (6.5)
20-24	75 512 (6.4)	89 304 (7.3)
25-29	77 178 (6.5)	84 411 (6.9)
30-34	68 702 (5.8)	72 177 (5.9)
35-39	71 183 (6.0)	68 507 (5.6)
40-44	76 275 (6.4)	77 070 (6.3)
45-49	89 920 (7.6)	88 081 (7.2)
50-54	96 528 (8.1)	97 868 (8.0)
55-59	88 166 (7.4)	89 304 (7.3)
60-64	70 504 (5.9)	74 624 (6.1)
65-69	53 524 (4.5)	52 604 (4.3)
70-74	41 771 (3.5)	42 817 (3.5)
75-79	41 026 (3.5)	37 924 (3.1)
80-84	33 856 (2.9)	35 477 (2.9)
≥85	29 822 (2.5)	35 477 (2.9)
% Male sex in total population	47.6^c^	47.8^e^
Population count per census tract	120-14222^c^	NA
Range per census tract (mean [SD])		
Age, y	23.9-64.4 (42.7 [6.8])^c^	40.5^f^
% Male sex by census tract	21.9-73.9 (45.4 [6.1])^c^	49.2^f^
% Smoker	9.2-21.1 (15.5 [5.0])^g^	15.9^h^
% With diabetes	1.4-69.2 (15.3 [14.8])^i^	10^j^
Range per census tract (mean [SD]) {total population mean}		
Total cholesterol level, mg/dL	167.1-194.7 (183.4 [4.4]) {189.36}^i^	189^k^
Blood pressure, mm Hg	112.7-133.2 (121.0 [3.9]) {122.6}^i^	122^l^
HDL-C level, mg/dL	48.8-57.7 (52.2 [1.4]) {52.17}^i^	40-59^m^

Abbreviations: FRED, A Framework for Reconstructing Epidemiological Dynamics; HDL-C, high-density lipoprotein cholesterol; NA, not applicable; NHANES, National Health and Nutrition Examination Survey.

SI conversion factors: To convert HDL-C and total cholesterol to millimoles per liter, multiply by 0.0259.

^a^ Values are mean ranges per census tract, except those values that are specific to the total population. Population means are given for reference when applicable and are specific to Allegheny County, Pennsylvania, when such data were available.

^b^ Data from US Census Bureau.^30^

^c^ Data from synthetic population used by FRED.

^d^ Group quarters included 38 305 individuals and were not used in this study.

^e^ Percentage of male individuals in the US; data from Howden et al.^31^

^f^ Data from Data USA.^32^

^g^ Mean of values assigned by matching to an individual selected from NHANES based on demographic characteristics.

^h^ Percentage of smokers in Pennsylvania; data from Centers for Disease Control and Prevention.^33^

^i^ Data from claims collected for the Data Across Sectors for Health project combined with values assigned using the NHANES for population outside of that covered by claims data.

^j^ Data from Allegheny County Health Department.^34^

^k^ Data from Miller.^35^

^l^ Data from Wright et al.^36^

^m^ Data from Lab Tests Online.^37^

Linear regression of observed CVD death risk from expected CVD death risk to assess model fit yielded a slope close to 1 (0.94; 95% CI, 0.75-1.12; *P* < .001) and an intercept not statistically significantly different from 0 (0.0013; 95% CI, –0.0014 to 0.0041; *P* = .38). On the basis of these and other metrics that evaluated the linear regression fit (eAppendix 1 and eFigure 1-3 in the Supplement), we considered the risk equation for assessing CVD death rate to give an acceptable estimation of risk.^27^

**Figure 2.**
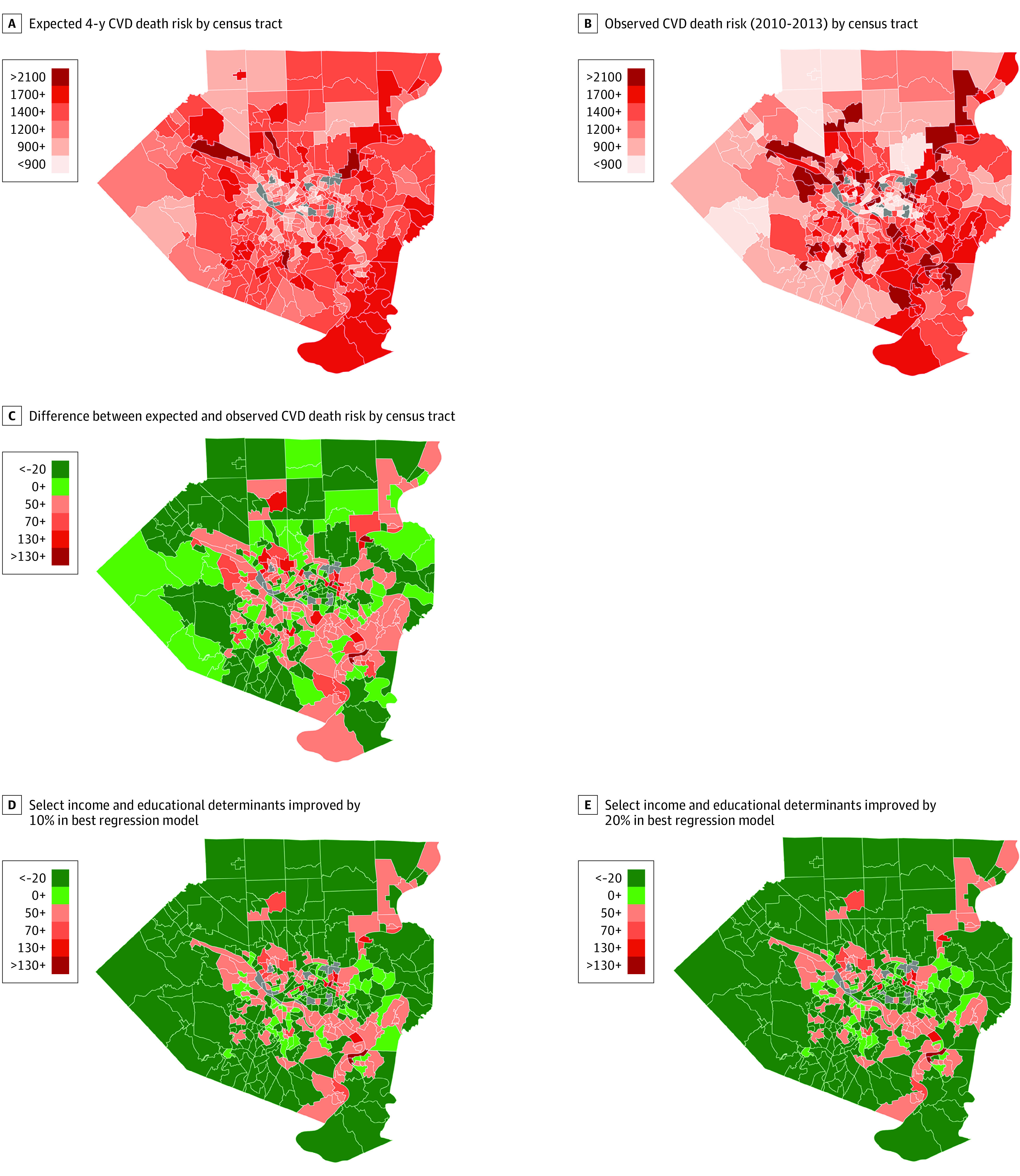
Expected, Observed, and Difference in per Census Tract 4-Year Cardiovascular Disease (CVD) Death Risk per 100 000 in Allegheny County, Pennsylvania In panels A and B, the scale refers to number of deaths per 100 000 persons per census tract. The gray areas were not included in the analysis because the population was too small for personally nonidentifiable values to be provided, so most data were missing. In panels C to E, green indicates less observed vs expected CVD death risk, and red indicates greater observed vs expected CVD death risk. The scales show excess deaths over expected deaths per 100 000 persons per census tract; negative values indicate greater risk, and positive values indicate less risk.

Observed 4-year rate of CVD death risk ranged from 220 to 6760 per 100 000 persons, with a mean (SD) of 1480 (592) per 100 000 persons (Figure 2A). Expected 4-year rate of CVD death risk per census tract ranged from 420 to 2450 per 100 000 persons, with a mean (SD) of 1440 (295) per 100 000 persons (Figure 2B). The difference between observed and expected risk ranged from –4810 to 1150 per 100 000 persons, with a mean (SD) of –40 (523) per 100 000 persons, in which the negative values indicated greater observed vs expected death risk and the positive values indicated less observed vs expected death risk (Figure 2C). Observed CVD death risk exceeded the expected CVD death risk in 166 of 348 census tracts (48%). Those census tracts represented 37% of total county population (443 188 of 1 188 112 individuals). The difference between expected and observed rates was not randomly distributed among census tracts (global univariate Moran *I*, 0.272; pseudo *P* = .001 with 999 permutations; *z* = 9.89) (eFigure 4 in the Supplement). Some census tracts with excess cardiovascular mortality were spatially contiguous (Figure 2C; eFigure 4C in the Supplement).

Most individual social determinants were statistically significantly associated with excess CVD death risk in univariate analyses by correlation analysis and linear regression (Table 2 and eTables 2 and 3 in the Supplement). The difference between expected and observed CVD death risk was most highly correlated with the percentage of households receiving food stamps (Pearson *r* = –0.488; *P* < .001), the percentage of households living below the federal poverty level (Pearson *r* = –0.406; *P* < .001), the poverty index (Pearson *r* = –0.422; *P* < .001), education-based social determinants (percentage of high school graduates: Pearson *r* = 0.492, *P* < .001; percentage of college graduates: Pearson *r* = 0.461, *P* < .001), and the median household income (Pearson *r* = 0.466; *P* < .001) (eTable 2 and eFigure 5 in the Supplement). Variables that did not reach statistical significance included the median age per census tract, the percentage of individuals living below the federal poverty level, the neighborhood walk score, the number of supermarkets per census tract, and the number of fast food restaurants per census tract.

**Table 2.** Correlation Between Expected and Observed Cardiovascular Disease Death Risk and Social and Biological Variables

Determinant	Univariate regression result		Income model		Income and education model		Biological model		Combined social and biological model	
	Regression slope (95% CI)	*P* value	Regression slope (95% CI)	*P* value	Regression slope (95% CI)	*P* value	Regression slope (95% CI)	*P* value	Regression slope (95% CI)	*P* value
Percentage of households receiving food stamps	−0.02 (−0.02 to −0.01)	<.001	−0.02 (−0.02 to −0.01)	<.001	−0.01 (−0.02 to −0.01)	<.001	NA	NA	−0.02 (−0.02 to −0.01)	<.001
Percentage without jobs	−0.02 (−0.03 to −0.016)	<.001	0.02 (0.01 to 0.03)	.0016	0.02 (0.01 to 0.03)	<.001	NA	NA	0.02 (0.012 to 0.035)	<.001
Median household income	1.0 × 10^−7^ −(8.0 × 10^−8^ to 1.20 × 10^−7^)	<.001	5.2 × 10^−8^(2.5 × 10^−8^ to 7.9 × 10^−7^)	<.001	3.6 × 10^−8^ (7.1 × 10^−9^ to 6.5 × 10^−8^)	<.0145	NA	NA	−2.6 × 10^−10^ (−3.1 × 10^−8^ to 3.1 × 10^−8^)	.99
Percentage with high school educational level	0.05 (0.04 to 0.06)	<.001	NA	NA	0.03 (0.01 to 0.04)	<.001	NA	NA	0.02 (0.01 to 0.03)	.006
Percentage with diabetes	NA	NA	NA	NA	NA	NA	−0.03 (−0.05 to −0.01)	.002	−0.01 (−0.03 to 0.01)	.36
Percentage with hyperlipidemia	NA	NA	NA	NA	NA	NA	0.04 (0.03 to 0.06)	<.001	0.02 (0.004 to 0.037)	.02
Percentage with hypertension,	NA	NA	NA	NA	NA	NA	−0.03 (−0.05 to −0.01)	.007	−0.02 (−0.044 to −0.001)	.04
High level of particulate matter in the census tract	−0.005 (−0.008 to −0.003)	<.001	NA	NA	NA	NA	NA	NA	NA	NA
Percentage with house in poor condition	−0.05 (−0.06 to −0.03)	<.001	NA	NA	NA	NA	NA	NA	NA	NA
Percentage of households renting	−0.007 (−0.01 to −0.005)	<.001	NA	NA	NA	NA	NA	NA	NA	NA
Percentage with college education	0.012 (0.010 to 0.015)	<.001	NA	NA	NA	NA	NA	NA	NA	NA
Individuals living below federal poverty level	−2 × 10^−3^ (−5 × 10^−3^ to 1 × 10^−3^)	.22	NA	NA	NA	NA	NA	NA	NA	NA
Percentage without insurance	−0.04 (−0.05 to −0.03)	<.001	NA	NA	NA	NA	NA	NA	NA	NA
Percentage of vacant houses in the census tract	−0.04 (−0.05 to −0.03)	<.001	NA	NA	NA	NA	NA	NA	NA	NA
Neighborhood walk score	−1 × 10^−5^ (−4 × 10^−5^ to 1 × 10^−5^)	.28	NA	NA	NA	NA	NA	NA	NA	NA
Percentage of households living below federal poverty level	−0.016 (−0.020 to −0.013)	<.001	NA	NA	NA	NA	NA	NA	NA	NA
Poverty index	−8 × 10^−4^ (−9 × 10^−4^ to −6 × 10^−4^)	<.001	NA	NA	NA	NA	NA	NA	NA	NA
No. of supermarkets per census tract	5 × 10^−4^ (−2 × 10^−4^ to 1.3 × 10^−3^)	.16	NA	NA	NA	NA	NA	NA	NA	NA
No. of fast food restaurants per census tract	1 × 10^−4^ (2 × 10^−5^ to 2.5 × 10^−4^)	.02	NA	NA	NA	NA	NA	NA	NA	NA
Percentage of households with no vehicle	−0.02 (−0.022 to −0.013)	<.001	NA	NA	NA	NA	NA	NA	NA	NA
Homicide counts per census tract	−3 × 10^−4^ (−5 × 10^−4^ to −2 × 10^−4^)	<.001	NA	NA	NA	NA	NA	NA	NA	NA
Median age	−8 × 10^−5^ (−2 × 10^−4^ to −9 × 10^−7^)	.05	NA	NA	NA	NA	NA	NA	NA	NA
Percentage with obesity	−0.02 (−0.021 to −0.01)	<.001	NA	NA	NA	NA	NA	NA	NA	NA

Abbreviation: NA, not applicable.

Some social determinants were highly correlated with each other (eTable 4 and eFigure 6 in the Supplement). Education-based measures (percentage of high school graduates and percentage of college graduates) correlated with income-based variables (median income [high school graduates: Pearson *r* = 0.689; college graduates: Pearson *r* = 0.721], the percentage of households receiving food stamps [high school graduates: Pearson *r* = –0.758; college graduates Pearson *r* = –0.656], and the percentage of households living below the federal poverty level [high school graduates: Pearson *r* = –0.710; college graduates: Pearson *r* = –0.452]) as well as the percentage without jobs (high school graduates: Pearson *r* = –0.639; college graduates: Pearson *r* = –0.561), the percentage without health insurance (high school graduates: Pearson *r* = –0.567; college graduates: Pearson *r* = –0.587), the percentage of households without a vehicle (high school graduates: Pearson *r* = –0.637; college graduates: Pearson *r* = –0.338), and the percentage of vacant houses in the census tract (high school graduates: Pearson *r* = –0.522; college graduates: Pearson *r* = –0.537). Obesity rate also correlated with these variables. Limited correlation with other social determinants was found for walk score, median age per census tract, and food desert markers.

**Figure 3.**
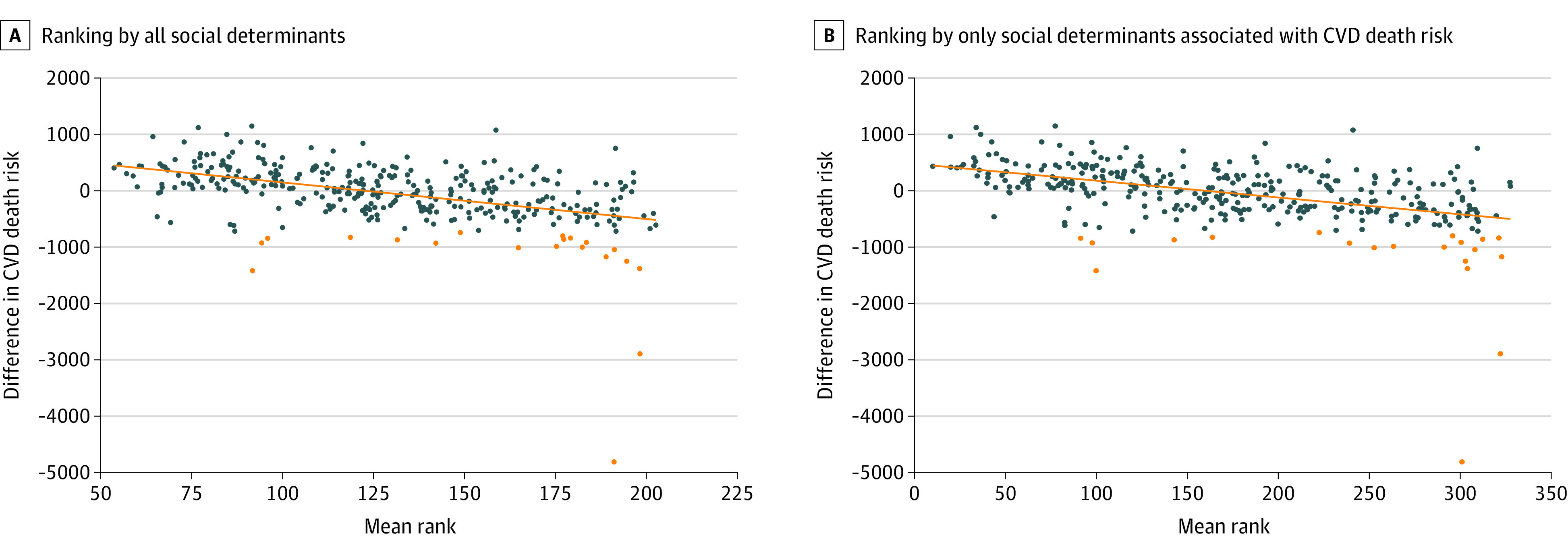
Plot of Difference in 4-Year Cardiovascular Disease (CVD) Death Risk by Mean Rank of Social Determinants per Census Tract Census tracts (circles) were ranked for level of each social determinant, and mean ranks were calculated for each census tract to get an overall ranking. Twenty census tracts with greatest excess in CVD death risk are plotted in orange. Social determinants associated with CVD death risk include percentage of high school graduates, percentage with a college degree, percentage with food stamps, percentage living below federal poverty level, percentage with obesity, median income, percentage of households with no vehicle, percentage without jobs, percentage without insurance, and percentage of vacant houses in the census tract. Regression line is in orange.

Mean rank of all social determinants for each census tract was negatively correlated with CVD risk difference (regression slope, –0.00005; 95% CI, –0.000057 to –0.000038; *P* < .001) (Figure 3A). When we included in ranking only the social determinants that were correlated with CVD risk difference, we found that the correlation was similar (regression slope, –0.00003; 95% CI, –0.000036 to –0.000024; *P* < .001) (Figure 3B). Twenty census tracts with greatest CVD risk difference did not cluster by mean social determinant rank for all social determinants or significantly correlated social determinants (Figure 3).

In the multivariate analysis including income-based social determinants of health (percentage of households receiving food stamps, percentage of households living below the federal poverty level, median income, poverty index, percentage without jobs, percentage of individuals living below the federal poverty level, and percentage without insurance), only the percentage of households receiving food stamps and the percentage without jobs were statistically significant variables. Stepwise elimination of nonsignificant variables improved model fit and resulted in a best selection of income variables, including the median income (regression slope, 5.2 × 10^−8^ [95% CI, 2.5 × 10^−8^ to 7.9 × 10^−7^]; *P* = 0.0016), the percentage of households receiving food stamps (regression slope, −0.02 [95% CI, −0.02 to −0.01]; *P* < .001), and the percentage without jobs (regression slope, 0.02 [95% CI, 0.01-0.03]; *P* < .001) (adjusted *R^2^*, 0.28; *P* < .001; residual SE, 0.004) (Table 2). Addition of the educational variable (percentage of high school graduates per census tract) improved model fit (adjusted *R^2^*, 0.30; *P* < .001; residual SE, 0.004) (Table 2). Variables associated with housing condition, vehicle access, particulate matter, walk score, smoking, and obesity were not significant and/or decreased model fit. Residual analysis for fit of linear models of difference between expected and observed 4-year CVD death risk by social determinants of health and risk factors associated with disease indicated that the models were robust (eFigure 7 in the Supplement).

In multivariate analysis, biological variables alone (diabetes, hypertension, and hyperlipidemia) accounted for approximately 27% of the variation in difference in CVD death risk (adjusted *R^2^*, 0.27; *P* < .001; residual SE, 0.004) (Table 2). Model fit was improved when biological variables were added to the best social determinant model (adjusted *R^2^*, 0.35; *P* < .001; residual SE, 0.004) (Table 2); however, in this model, median income was no longer statistically significant.

The potential outcome of changing social determinants was tested by modifying the determinant value by census tract in the income and education model (Table 2; eTable 3 in the Supplement). In original data, 166 of 348 census tracts (48%) had a negative difference between observed and expected CVD death risk, indicating that the observed risk was higher than the expected risk. Improving the determinants by applying the effect of increasing positive determinants and decreasing negative determinants resulted in a lower number of census tracts with higher estimated mortality than expected (10% improvement: 124 census tracts with negative difference, census tract population count of 329 294, and 28% of total county population; 20% improvement: 111 census tracts with negative difference, census tract population count of 303 485, and 26% of total county population) (Figure 2D-E). Applying the same level of improvement in social determinant value to the combined social and biological model (Table 2; eTable 3 in the Supplement) yielded a similar decrease in risk (10% improvement: 124 census tracts with negative difference, census tract population count of 345 766, and 29% of total county population; 20% improvement: 105 census tracts with negative difference, census tract population count of 284 279, and 24% of total county population).

## Discussion

To explore the distribution of excess CVD death risk and its correlation with social determinants of health in Allegheny County, Pennsylvania, we created a geographically and demographically realistic semisynthetic population and used local insurance claims and local social determinant values. Income and education–based social determinants correlated at the census tract level with excess CVD death risk, a finding that is consistent with the results of other studies.^13,17,38^ Census tracts with excess CVD risk were sometimes contiguous, although large differences between adjacent census tracts were also found. This study also modeled the estimated effect of improving social determinants of health and risk factors associated with disease. Although improvement did decrease the modeled CVD death rates enough to bring them to the expected level of risk in some census tracts, higher risk remained in many census tracts. Whether the highest levels of CVD mortality risk can be brought to median risk by achievable levels of social determinant mitigation is unknown.

Results of this study show that some local areas have much worse outcomes than expected for CVD death risk, whereas other areas have better outcomes. This difference in excess risk correlates with disparities in income and education but the correlation among the social determinants themselves suggests that addressing a combination of factors may be helpful in decreasing risk. Many of these variables work over long periods. For example, educational level shapes an individual’s financial trajectory, employment opportunities, and access to insurance over their lifetime. These outcomes will be cumulative for the individual and will be enhanced over generations. Factors that interact synergistically will likely affect cardiovascular health, and the biological factors that shape CVD death risk are, in turn, shaped by the societal context in which the individual exists.^39^ Interventions for social risk factors can be applied at an early age, to prevent the development of habits that could increase lifetime risk. Initiatives to increase the uptake or use of available health services and programs may be as important as implementation of the programs and services, given that an intervention can only succeed if the groups for whom it is intended use it.

Several possible theories might explain the existence of regions with high levels of excess mortality risk, such as the 2 outlier regions found in this study. A local area may have genetics- or race/ethnicity-based reasons for excess risk, although this explanation was not reflected in the population of the outlier regions in this study, which were not more racially segregated than other census tracts in the region. Excess risk may reflect the history of the area. In Allegheny County, the decline in the manufacturing industry may have led to the widespread, long-term unemployment among groups of people who were dependent on the industry and therefore lived near those factories, creating pockets of areas with low income and underinsurance. It is likely that a specific factor plays a role in the overall situation of the individual, but no single characteristic is solely responsible for a health outcome.

The results suggest that to decrease high rates of CVD death risk, interventions for biological risk factors should be combined with interventions targeted at social factors. Long-term, community-based interventions for biological and behavioral risk factors have been associated with decreased rates of CVD.^40^ A number of studies have found better health outcomes and lower health care costs after social and environmental factors are addressed.^41,42^ An analysis of studies evaluating such interventions found that improvement in health outcomes and health care costs was frequently associated with programs supporting the need for housing, nutrition, and income.^41^ Interventions to produce better housing quality and to reduce educational and income disparities could play a role in lowering excess CVD death risk. Interventions at the neighborhood level have proven to be successful in changing behaviors that lead to health risks.^42^

The method we used to identify excess mortality risk may be used in targeting resources and identifying the underlying factors associated with that excess risk. One strategy is to tailor interventions to locations or groups with the greatest risk, particularly when resources are limited. Another strategy is to identify areas with high excess risk to enable further investigation of other underlying factors or combinations of factors associated with the outcome. The excess risk method can be applied to myriad other conditions, such as diabetes and asthma.

Although large-scale aggregations of risk data may not provide sufficient discrimination to identify factors in increased risk, individual-level data may also be insufficient for this purpose given that such data cannot assess the implication of the environment at the population level.^43^ This situation argues for the use of a technique such as the one discussed here that provides data at a spatial granularity at which both individual and environmental risk factors can be evaluated. Census tracts are an ideal spatial unit for this type of analysis, not only because of their size but also because their boundaries are chosen to include economically homogeneous populations and because federal, state, and local programs are often targeted by census tracts.^44^

### Strengths and Limitations

This study has several strengths. A major strength was the novel creation of a biologically and geographically realistic semisynthetic population and the use of local data sets on multiple social determinants associated with CVD risk, data that are not commonly available in combination with a detailed local population. The spatially explicit method used for creating the synthetic population is generalizable to any location for which similar information is available. These populations form the basis of agent-based and other types of modeling projects. In addition, the concept of excess observed risk over expected risk can be used to investigate the reasons for that excess risk.

This study has several limitations. We assessed CVD death risk using an existing risk equation, which was chosen in part because most of the required variables could be distributed in the synthetic population from available sources. This risk equation gave results that were largely similar to real data on CVD death risk at the census tract level. The social determinants of health that we examined were highly correlated among themselves, making it difficult to identify the most important ones. Social determinant data were collected from a variety of sources and not at the same time point. Proving a causal connection between social determinants and health outcomes was not possible with the data, but the results provide support for the population-based association between social determinants and CVD death. The study was conducted with aggregate determinant data, and a full analysis of spatial autocorrelation was beyond its scope.

## Conclusions

This study found that spatially explicit modeling can aid in identifying which geographic locations might gain the most advantage from interventions. Estimating the potential outcome of interventions for large population groups is difficult and may require access to individual-level factors associated with disease, data that are rarely available. Creation of a semisynthetic population, in which members of a synthetic population are assigned plausible values for biological, social, and other variables using measured population data, is a logical method for estimating the potential outcome of interventions. This method can be applied to a variety of diseases for which data on incidence and associated factors are available.

## Notes

Supplement. **eMethods.** Description of Synthetic Population Creation **eAppendix 1.** Description and Evaluation of Algorithm Used for Prediction of Cardiovascular Disease Death Rate **eFigure 1.** Histogram of Difference Between Expected and Observed CVD Death Rate **eFigure 2.** Plot of Difference Between Expected and Observed Census Tract Level Cardiovascular Disease Death Risk **eFigure 3.** Results of Evaluation of Algorithm Used for Prediction of CVD Death Rate **eAppendix 2.** Sources, Description and Limitations of Social Determinants of Health Data **eTable 1.** Missing Data for Social Determinants of Health **eAppendix 3.** Methods for Calculation of Univariate Global Moran’s I and Local Indicators of Spatial Association for Evaluation of Spatial Clustering **eFigure 4.** Spatial Autocorrelation of Difference Between Expected and Observed CVD Death Rate **eTable 2.** Correlation of Social Determinants With Difference Between Expected and Observed Cardiovascular Disease Death Rate **eFigure 5.** Plots of Difference Between Expected and Observed Cardiovascular Disease (CVD) Death Rates and Representative Social Determinants of Health **eTable 3.** Linear Regression Median and Interquartile Range for Univariate and Combined Models of Individual Level Risk Difference Between Expected and Observed Cardiovascular Disease Death Risk **eTable 4.** Correlation of Social Determinants by Pearson’s Correlation Coefficient **eFigure 6.** Pairwise Plots of Selected Representative Social Determinants by Census Tract **eFigure 7.** Residual Analysis for Fit of Linear Models of Difference Between Expected and Observed Four-Year Cardiovascular Disease Death Risk by Social and Disease Determinants **eReferences**
