# Supplementary material for: Development of a Synthetic Population Model for Assessing Excess Risk for Cardiovascular Disease Death
Source: JAMA Netw Open. 2020 Sep 1;3(9):e2015047. doi: 10.1001/jamanetworkopen.2020.15047 (PMC7489828; doi:10.1001/jamanetworkopen.2020.15047)
Supplement: Supplement. — eMethods. Description of Synthetic Population Creation eAppendix 1. Description and Evaluation of Algorithm Used for Prediction of Cardiovascular Disease Death Rate eFigure 1. Histogram of Difference Between Expected and Observed CVD Death Rate eFigure 2. Plot of Difference Between Expected and Observed Census Tract Level Cardiovascular Disease Death Risk eFigure 3. Results of Evaluation of Algorithm Used for Prediction of CVD Death Rate eAppendix 2. Sources, Description and Limitations of Social Determinants of Health Data eTable 1. Missing Data for Social Determinants of Health eAppendix 3. Methods for Calculation of Univariate Global Moran’s I and Local Indicators of Spatial Association for Evaluation of Spatial Clustering eFigure 4. Spatial Autocorrelation of Difference Between Expected and Observed CVD Death Rate eTable 2. Correlation of Social Determinants With Difference Between Expected and Observed Cardiovascular Disease Death Rate eFigure 5. Plots of Difference Between Expected and Observed Cardiovascular Disease (CVD) Death Rates and Representative Social Determinants of Health eTable 3. Linear Regression Median and Interquartile Range for Univariate and Combined Models of Individual Level Risk Difference Between Expected and Observed Cardiovascular Disease Death Risk eTable 4. Correlation of Social Determinants by Pearson’s Correlation Coefficient eFigure 6. Pairwise Plots of Selected Representative Social Determinants by Census Tract eFigure 7. Residual Analysis for Fit of Linear Models of Difference Between Expected and Observed Four-Year Cardiovascular Disease Death Risk by Social and Disease Determinants eReferences [file jamanetwopen-e2015047-s001.pdf]

Supplementary Online Content

Krauland MG, Frankeny RJ, Lewis J, et al. Development of a synthetic population model for assessing excess risk for cardiovascular disease death. *JAMA Netw Open*. 2020;3(9):e2015047. doi:10.1001/jamanetworkopen.2020.15047

eMethods. Description of Synthetic Population Creation

eAppendix 1. Description and Evaluation of Algorithm Used for Prediction of Cardiovascular Disease Death Rate

eFigure 1. Histogram of Difference Between Expected and Observed CVD Death Rate

eFigure 2. Plot of Difference Between Expected and Observed Census Tract Level Cardiovascular Disease Death Risk

eFigure 3. Results of Evaluation of Algorithm Used for Prediction of CVD Death Rate

eAppendix 2. Sources, Description and Limitations of Social Determinants of Health Data

eTable 1. Missing Data for Social Determinants of Health

eAppendix 3. Methods for Calculation of Univariate Global Moran's I and Local Indicators of Spatial Association for Evaluation of Spatial Clustering

eFigure 4. Spatial Autocorrelation of Difference Between Expected and Observed CVD Death Rate

eTable 2. Correlation of Social Determinants With Difference Between Expected and Observed Cardiovascular Disease Death Rate

eFigure 5. Plots of Difference Between Expected and Observed Cardiovascular Disease (CVD) Death Rates and Representative Social Determinants of Health

eTable 3. Linear Regression Median and Interquartile Range for Univariate and Combined Models of Individual Level Risk Difference Between Expected and Observed Cardiovascular Disease Death Risk

eTable 4. Correlation of Social Determinants by Pearson's Correlation Coefficient

eFigure 6. Pairwise Plots of Selected Representative Social Determinants by Census Tract

eFigure 7. Residual Analysis for Fit of Linear Models of Difference Between Expected and Observed Four-Year Cardiovascular Disease Death Risk by Social and Disease Determinants

eReferences

This supplementary material has been provided by the authors to give readers additional information about their work.

©2020 Krauland MG et al. *JAMA Network Open*

eMethods. Description of Synthetic Population Creation

A synthetic population with demographics and disease characteristics was built from the synthetic population used by the FRED modeling and simulation platform (Figure 1).[1] The FRED population was generated by a census-based iterative proportional fitting methodology that results in a geospatially realistic population that accurately represents the demographics and household structure of the US population.[2]

Counts of insured population with evidence of specific conditions by claims for Type II diabetes, hyperlipidemia, hypertension, and combinations of these conditions was provided by three major insurance organizations in the Allegheny County area. The covered population included private insurance, Medicare and Medicaid. Eligible enrollees were Allegheny County residents, enrolled for at least 90 continuous days under one of the three health plans in the year 2015 (Jan 1 – Dec 31). Medical claims data was provided for 30-86% of population in 97.2% of census tracts. Eligible enrollees varied by census tract but overall accounted for approximately 60 percent of the Allegheny County population. Percent of persons in a given census tract who were insured by each insurer or by the three insurers overall was calculated, but causes of lack of insurance are not available. At least one census tract with low reported percentage of insurance coverage is the location of 2 major universities and therefore is residence of a large number of students from other locations, who may have insurance through other carriers, but it is not possible to quantitate that from the available data. Since the insurers providing data included the largest Medicaid insurance provider in the area, Medicaid enrollees are presumed to be represented adequately in the claims data. Other reasons for low coverage in a census tract include cost of insurance, perceived lack of need for insurance, distrust of the system and lack of access to or knowledge about availability of insurance programs are possible but the level of these or other factors on insurance uptake at the census tract level are beyond the scope of this study. Further, some proportion of the population likely had insurance with other insurers who cover small portions of the county population and this may not be distributed evenly the population. This could affect the results and is a limitation of the study

Census tract levels for each disease were stratified by gender and age range category (1-17, 18-44, 45-64, 65-84, and ages 85+).

Disease claim counts were used to assign levels of diabetes, hypertension and hyperlipidemia and combinations of these conditions to the FRED population on a census tract basis. It is not possible to connect a diagnostic variable (such as diabetes) directly to an individual in the claims data. When the number of agents per census tract exceeded the population covered by insurer data, agent diabetes, hypertension and hyperlipidemia status were assigned by randomly drawing an individual with matching demographics from the National Health and Nutrition Examination Survey (NHANES).[3] Plausible values for individual height, cholesterol, high density lipoproteins, blood pressure and history of stroke and prior myocardial infarction were obtained from NHANES, by matching to the synthetic population's agent-level demographics and disease status. When agent diabetes, hypertension and hyperlipidemia status were assigned from NHANES, the NHANES values for individual height, cholesterol, high density lipoproteins, blood pressure and history of stroke and prior myocardial infarction from that NHANES individual were used to assign those variables to that agent. Data from the National Health Interview Survey was used to assign smoking status to agents based on demographics.[4] Rates were obtained by summing counts per tract and dividing by population. Each agent was assigned a five-year risk of death due to CVD using a published risk equation (see eAppendix 1).

©2020 Krauland MG et al. *JAMA Network Open*

eAppendix 1. Description and Evaluation of Algorithm Used for Prediction of Cardiovascular Disease Death Rate

To predict risk of death from cardiovascular disease, this study used a risk score that was developed using data from eight randomized clinical trials for treatment of hypertension.[5] Development of the risk score related individual characteristics to risk of death from cardiovascular disease using a multivariate Cox model. A risk score was developed from 11 factors: age, sex, systolic blood pressure, serum total cholesterol concentration, height, serum creatinine concentration, cigarette smoking, diabetes, left ventricular hypertrophy, history of stroke, and history of myocardial infarction. The risk score is an integer, with points added for each factor according to its association with risk. This risk score algorithm was chosen in part because the majority of individual level characteristics needed for the prediction were available in the FRED synthetic population, could be added from the insurer claims data available for this project or could be distributed in the population in a realistic way by choosing random similar individuals from NHANES or NHIS. Creatinine values were not available so 2 points was added to the risk score for all agents as suggested by the risk calculator developers. Left ventricular hypertrophy was also not available so was not used in the calculation. Risk was scaled to four years to match data and was summed over each census tract. Agents 18 years old or younger were assigned zero risk.

Difference between expected and observed CVD death risk was approximately normally distributed by Shapiro-Wilk normality test (W = 0.99219, p-value = 0.06335) after removal of 2 outliers (eFigure1). Average difference between expected and actual CVD death risk was close to 0 (-40, SD 524) and approximately evenly distributed around 0 but with 2 notable outliers (eFigure2). Linear regression was used to evaluate the reliability of the algorithm used for prediction of cardiovascular disease (CVD) death risk.[6] Regression of observed CVD death rate risk from expected rate gave an intercept not significantly different from 0 (0.0013, CI [-0.0014, 0.0041], p=0.384) and slope close to 1 (0.94, CI [0.75, 1.12], p <0.001), with an adjusted R-squared of 0.214 and F-statistic: 95.87 on 1 and 348 DF (p-value: < 0.001). Plot of residuals versus fitted values did not show any pattern (eFigure 3A) and normal Q-Q plot showed residuals were normally distributed, with the exception of 2 outliers (eFigure 3B). Scale-location plot indicated limited unequal variance (eFigure 3C). All points were within curved lines in plot of residuals vs leverage, although the outliers were close to the 0.5 line (eFigure 3D). Based on these metrics, this method was considered to provide an acceptable estimate of population risk.

©2020 Krauland MG et al. *JAMA Network Open*

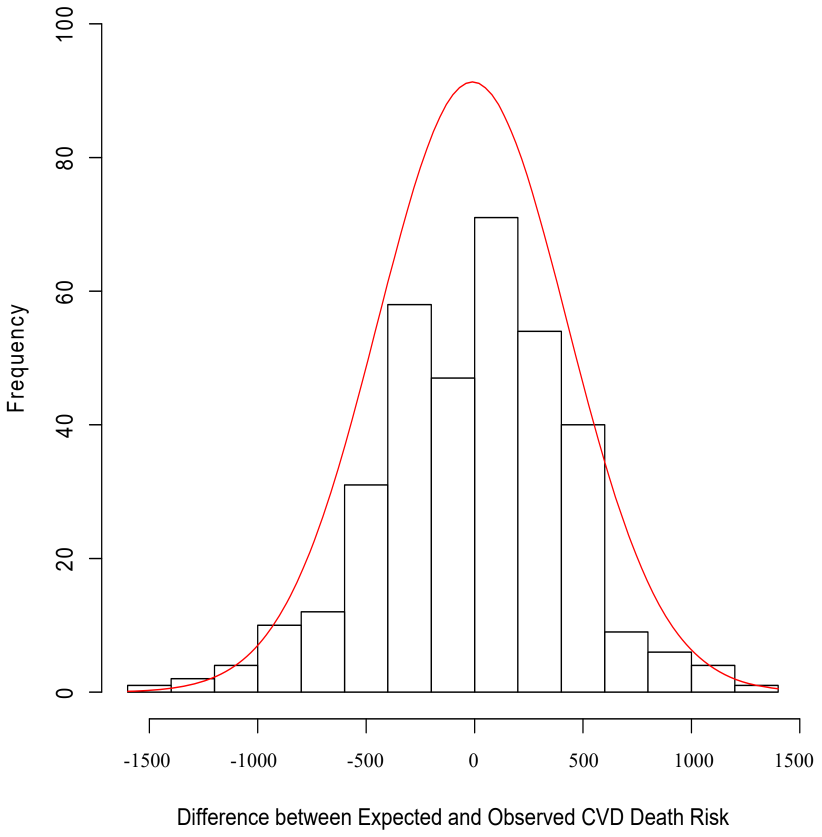

eFigure 1. Histogram of Difference Between Expected and Observed CVD Death Rate

Difference between expected and observed CVD death risk per 100,000 was approximately normally distributed, after removal of 2 outliers. Normal curve plotted in red.

©2020 Krauland MG et al. *JAMA Network Open*

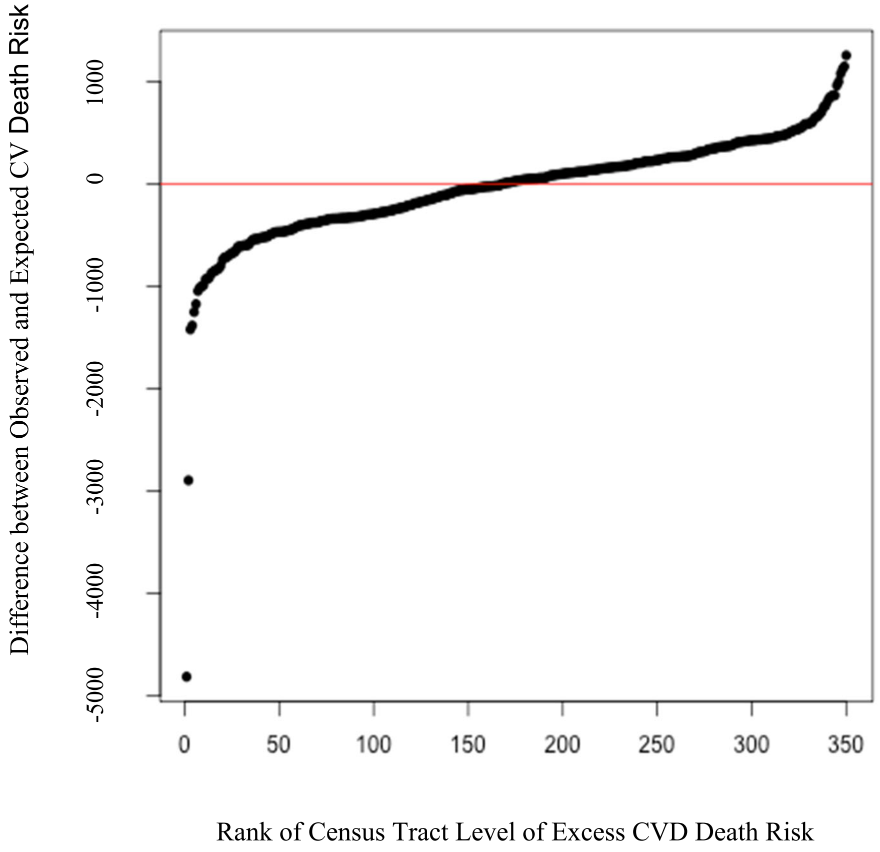

eFigure 2. Plot of Difference Between Expected and Observed Census Tract Level Cardiovascular Disease Death Risk

Data was ranked lowest to highest value. Dots represent difference between expected and observed death risk per 100,000 population. Red line is 0 difference.

©2020 Krauland MG et al. *JAMA Network Open*

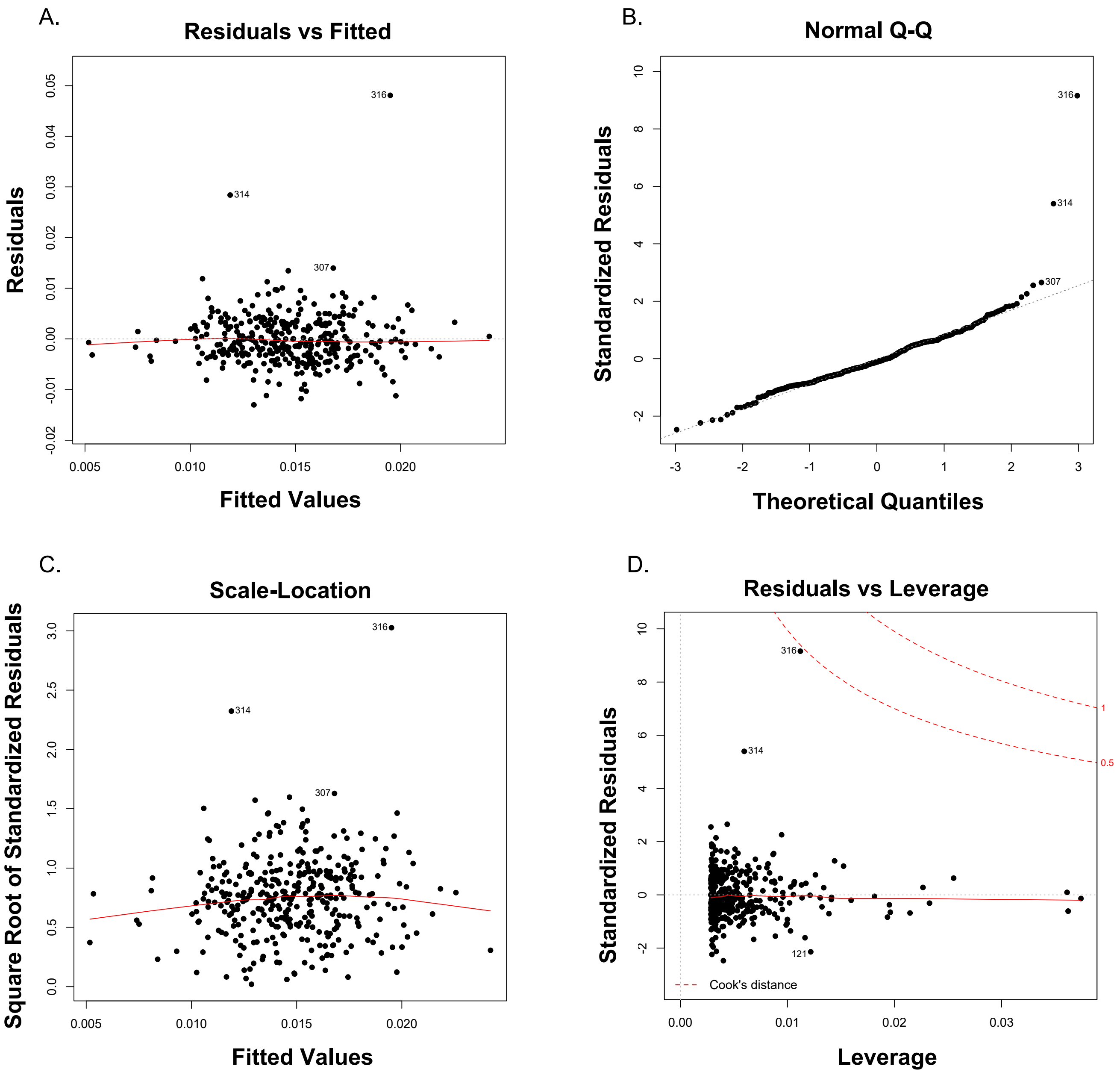

eFigure 3. Results of Evaluation of Algorithm Used for Prediction of CVD Death Rate

Regression of census-tract level observed cardiovascular death risk vs. cardiovascular death risk predicted using a cardiovascular disease death risk prediction algorithm and synthetic population variables to assess fit of predicted risk. A. Plot of residuals versus fitted values did not show any pattern. B. Normal Q-Q plot showed residuals were normally distributed, with the exception of 2 outliers. C. Scale-location plot indicated limited unequal variance. D. All points were within curved lines in plot of residuals vs leverage, although the outliers were close to the 0.5 line.

eAppendix 2. Sources, Description and Limitations of Social Determinants of Health Data

Social determinants of health data for the Data Across Sectors for Health (DASH) project were collected by the Allegheny County Health Department (ACHD) and partners of the Allegheny County Data Sharing Alliance for Health (ACDSAH). Data used in this study is hosted by the Western Pennsylvania Regional Data Center at the University of Pittsburgh Center for Social & Urban Research (https://data.wprdc.org/dataset?tags=ACHD+DASH) and maintained and updated by the ACHD. Social determinants investigated in this study were related to education (percent high school graduate, percent college graduate), income (percent unemployed, percent of individuals below poverty level, percent households with no vehicle, poverty index, median household income, percent households with food stamps, percent households below poverty level), access to food (number of supermarkets per tract, number of fast food restaurants per tract), housing condition and neighborhood safety (percent vacant houses, walk score, percent households renting, percent houses in poor condition, particulate matter, homicide counts per tract), access to health care (percent uninsured) and personal characteristics (median age, obesity rate).
Census tracts for which a majority of determinants were missing were omitted from the study (n=5).

Data Description and Limitations
Data was collected in 2016-2017 but in some cases was for prior periods, as noted. Data was provided at percent or counts per census tract and was not available at higher granularity. Two data variables had a large proportion of missing data (eTable 1) and it was not possible to either obtain those missing values or to determine what caused the data to be missing. Level of missing data was low for the most part (of 20 determinants, 17 had 5 or fewer tracts with missing data). Different variables were not generally missing for the same tracts.
Percent vacant property estimates were produced by the US Postal Service. Vacant properties data is routinely collected by mail carriers on addresses no longer receiving mail due to vacancy and is reported quarterly at census tract geographies in the United States along with counts of total mailing addresses. Data used in this study was aggregated to Allegheny County census tracts.

Allegheny County Particulate Matter 2.5 data was obtained from the U.S. Department of Environmental Protection's Environmental Justice Screen tool. Particulate Matter 2.5 levels were retrieved from National Air Toxics Assessment 2011 CMAQ-derived predicted PM2.5 data, census tract specific estimates (https://www.epa.gov/hesc/rsig-related-downloadable-data-files). Information is on the particulate matter concentration for Allegheny County that had a diameter greater or equal to 2.5 mm and is based on a combination of 2012 model and monitor data reported by the U.S. Environmental Protection Agency's Office of Air and Radiation. The data represents the mean PM 2.5 measurement in micrograms per cubic meter for each census tract in Allegheny County.

Location information for all Supermarkets and Convenience Stores in Allegheny County was produced using the Allegheny County Fee and Permit Data for 2016. Fee and permit data were used to generate number of fast food restaurants (restaurant that has more than one location in the county but without an alcohol permit) and number of restaurants per census tract (ACHD Fee and Permit data, 2016). Census tract level counts of Allegheny County Fast Food Establishments was obtained by exporting all chain restaurants without an alcohol permit from the County's Fee and Permit System. Chain restaurants capture both local and national chains (including locally owned national chains) so long as there is one or more establishments in operation within the County. While access to supermarkets and excess fast food establishments are believed to impact nutrition and therefore health, in this dataset that effect was not apparent. Most tracts had 0 or 1 supermarket and this study did not include analysis of access to transportation to supermarkets. Fast food establishments were concentrated in the downtown area, where a large number of individuals work but few reside, and in the university areas, where again there are many workers who reside elsewhere and additionally there are many students, who are less at risk for cardiovascular disease.
Poor Housing Conditions is an estimate of the percent of distressed housing units in each Census Tract and was prepared using data from the American Community Survey and the Allegheny County Property Assessment

database (https://data.wprdc.org/dataset/property-assessments). The estimate was produced by the Allegheny County Reinvestment Fund with the Allegheny County Department of Economic Development.
Obesity rates for each census tract were obtained from a published study.[7] Obesity rates for each Census Tract in Allegheny County were produced by estimates using statistical modeling techniques. The obesity rate of a demographically similar census tract was applied to similar ones in Allegheny County to compute an obesity rate.[7]
Census tract walk scores measure the walkability of any address using a patented system developed by the Walk Score company. Walk scores were produced by Walk Score (https://www.walkscore.com). For each 2010 Census Tract centroid, Walk Score analyzed walking routes to nearby amenities. Points were awarded based on the distance to amenities in each category. Amenities within a 5 minute walk (.25 miles) are given maximum points. A decay function is used to give points to more distant amenities, with no points given after a 30 minute walk. Walk Score also measures pedestrian friendliness by analyzing population density and road metrics such as block length and intersection density. Data sources include Google, Education.com, Open Street Map, the U.S. Census, Localeze, and places added by the Walk Score user community. While walking scores indicate the ability of residents to walk to amenities, probability of individuals having increased fitness levels by walking to them is highly variable.
Homicide counts were obtained from the Department of Human Services. Homicide counts were found to often be located at the hospital where an affected individual would have died, so this variable was considered non-informative.

The following census tract level data was obtained from the American Community Survey, US Census, American Factfinder: median age; educational attainment; employment status; Food Stamps/SNAP; and housing type.

eTable 1. Missing Data for Social Determinants of Health

Determinant	Number of Observations Missing (Percent missing)	Determinant	Number of Observations Missing (Percent missing)
Percent High School Graduates	0 (0)	Percent housing in poor condition	94 (27.0)
Percent College Graduate or Higher	0 (0)	Poverty Index	11 (3.2)
Number of Fast food restaurants	0 (0)	Particulate matter (Ppm2.5)	0 (0)
Percent households receiving foodstamps	5 (1.4)	Percent renters	0 (0)
Percent households below poverty level	5 (1.4)	Number of supermarkets	0 (0)
Homicides	2 (0.6)	Percent unemployed	3 (0.8)
Median age	0 (0)	Percent people below poverty level	56 (16.1)
Median income	1 (0.3)	Percent uninsured	1 (0.3)
Percent households with no vehicle	1 (0.3)	Percent vacant housing	1 (0.3)
Percent Obese	3 (0.8)	Walk score	0 (0)
Total census tracts	348		

©2020 Krauland MG et al. *JAMA Network Open*

eAppendix 3. Methods for Calculation of Univariate Global Moran's I and Local Indicators of Spatial Association for Evaluation of Spatial Clustering

We performed an analysis of Global Moran's I to assess spatial autocorrelation of difference between expected and observed CVD death risk at the census tract level.[8] Randomization with 999 permutations gave a pseudo p-value of 0.001, rejecting the null hypothesis that the distribution of difference was random in the county (eFigure 4, A and B). We further performed Local Indicators of Spatial Association (LISA) analysis to identify regions of clustering. Some areas of high-high and low-low clusters were identified, supporting the hypothesis that there was a degree of clustering of high and low risk census tracts within the county. Further analysis of spatial autocorrelation was beyond the scope of this study.

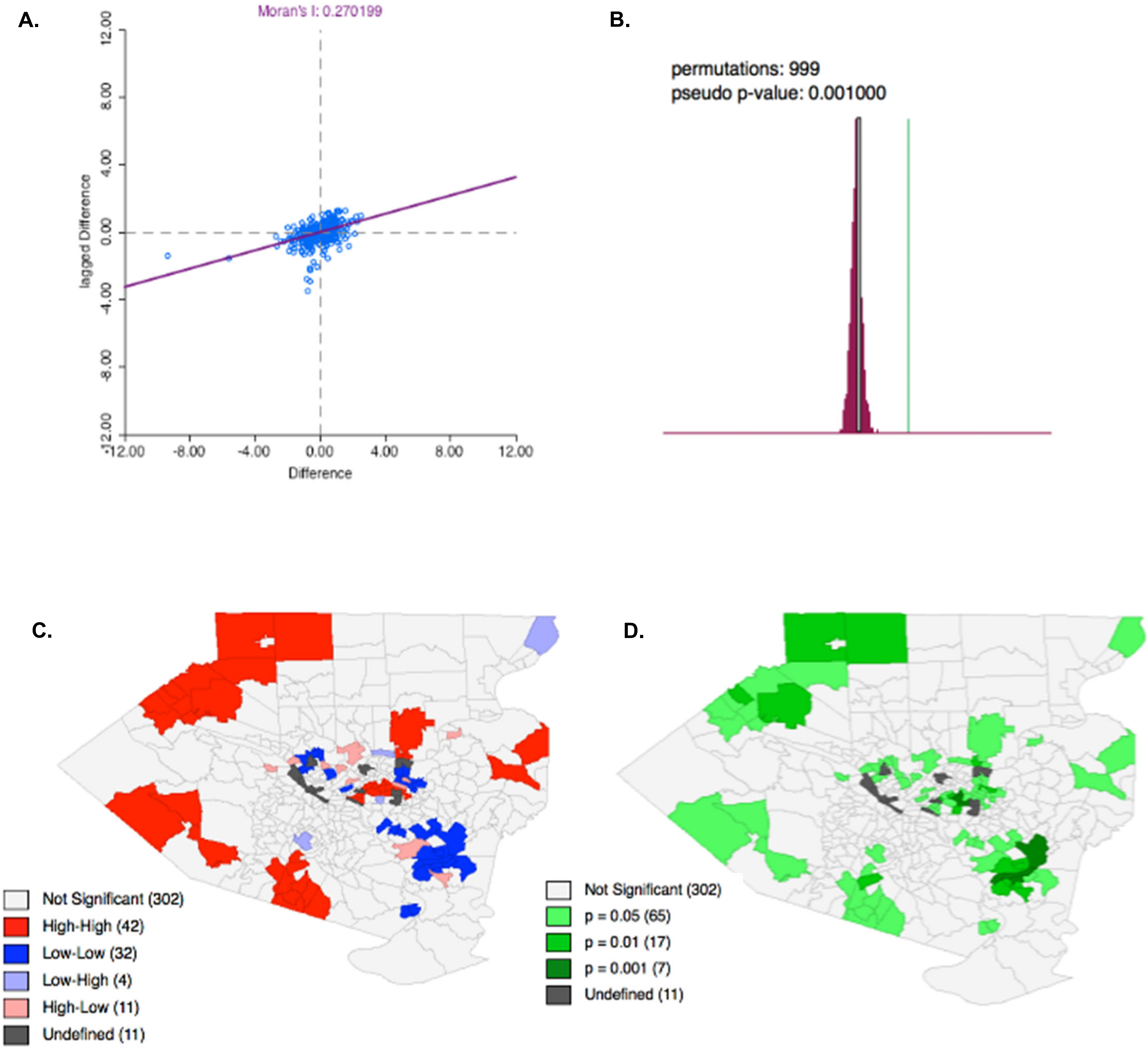

eFigure 4. Spatial Autocorrelation of Difference Between Expected and Observed CVD Death Rate

A. Univariate Global Moran's I. B. Univariate Global Moran's I significance. C. LISA Cluster Map of difference between predicted and actual cardiovascular disease death rate (Univariate Local Moran's I). D. Significance map of Univariate Local Moran's I of difference between predicted and actual cardiovascular disease death rate.

©2020 Krauland MG et al. *JAMA Network Open*

eTable 2. Correlation of Social Determinants With Difference Between Expected and Observed Cardiovascular Disease Death Rate

Determinant	Correlation[a]	Probability[b]
High school graduate (%)	0.492	**< 0.001**
College education (%)	0.461	**< 0.001**
Households with food stamps (%)	-0.488	**< 0.001**
Households below poverty level (%)	-0.406	**< 0.001**
Homicide counts	-0.304	**< 0.001**
Median age	-0.106	0.03
Obesity Rate (%)	-0.351	**< 0.001**
Particulate matter	-0.209	0.04
Houses in poor condition (%)	-0.392	**< 0.001**
Households renting (%)	-0.292	**< 0.001**
Unemployed (%)	-0.309	**< 0.001**
Individuals below poverty level (%)	-0.071	0.19
Uninsured (%)	-0.372	**< 0.001**
Vacant houses (%)	-0.355	**< 0.001**
Walk score	-0.058	0.44
Median household income	0.466	**< 0.001**
Poverty index	-0.422	**< 0.001**
Number supermarkets	0.075	0.12
Number fast food restaurants	0.127	0.01
Households with no vehicle (%)	-0.364	**< 0.001**

[a] Pearson's product-moment correlation

[b] Significant probabilities marked in bold

©2020 Krauland MG et al. *JAMA Network Open*

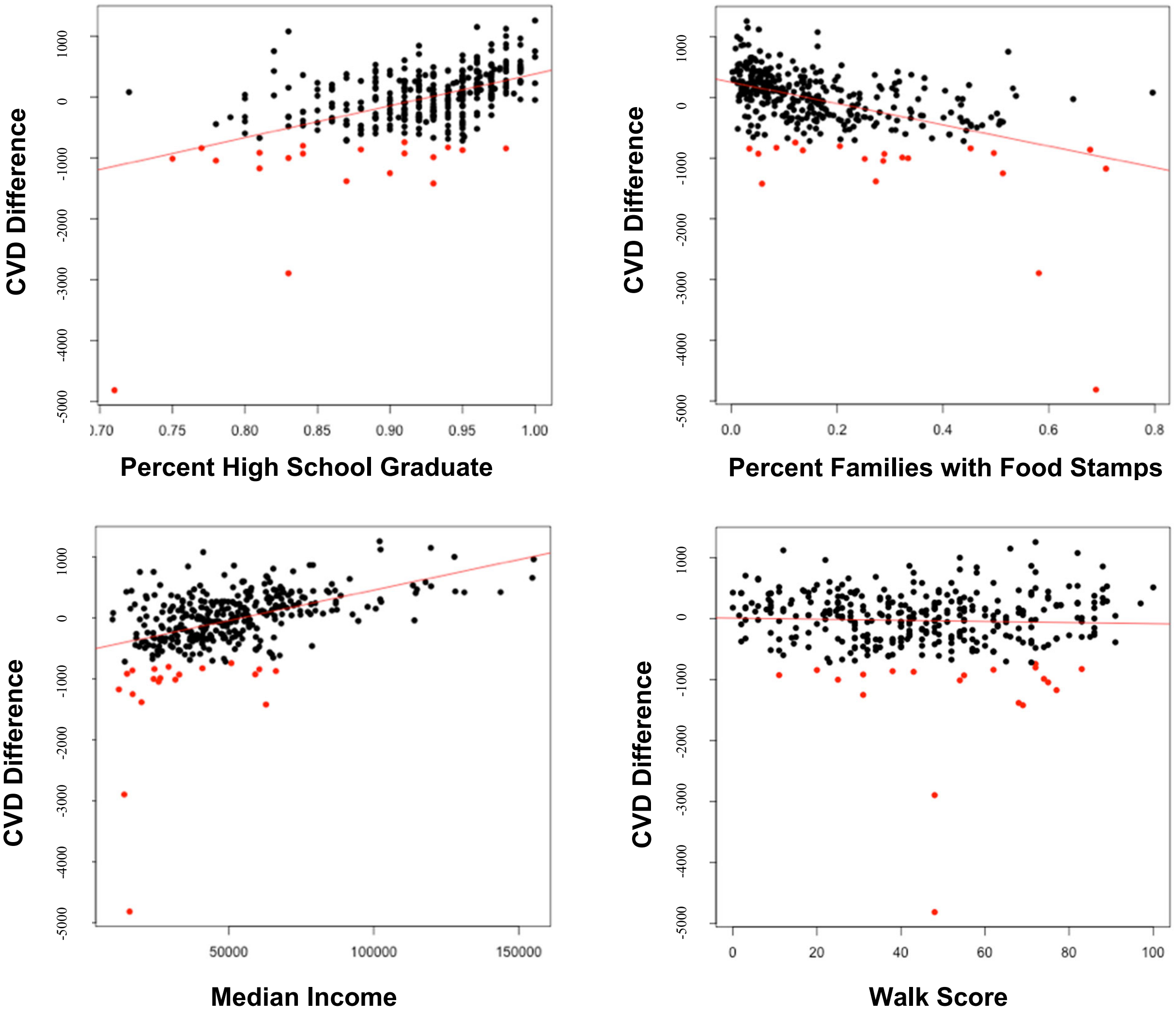

eFigure 5. Plots of Difference Between Expected and Observed Cardiovascular Disease (CVD) Death Rates and Representative Social Determinants of Health

Values of CVD difference below zero indicate excess observed disease over expected disease. Income related determinants had strong relationship with risk of death, while walk score showed no relationship. Twenty census tracts with greatest excess in cardiovascular disease death risk plotted in red. Regression line plotted in red.

©2020 Krauland MG et al. *JAMA Network Open*

eTable 3. Linear Regression Median and Interquartile Range for Univariate and Combined Models of Individual Level Risk Difference Between Expected and Observed Cardiovascular Disease Death Risk

Determinant	Univariate	Income Model	Income and Education Model	Biological Model	Combined Social and Biological Model
Households with food stamps (%)	0.000199 (-0.038598, 0.014130)	0.000238 (-0.036187, 0.012065)	0.000074 (-0.033458, 0.013442)	-	0.000033 (-0.033378, 0.011368)
Unemployed (%)	0.000277 (-0.047753, 0.012702)			-	
Median household income	0.000207 (-0.044137, 0.012207)			-	
High school graduate (%)	0.000063 (-0.036941, 0.015842)	-		-	
Diabetes	-	-	-	0.000080 (-0.042618, 0.011594)	
Hyperlipidemia	-	-	-		
Hypertension	-	-	-		
Particulate matter	0.000522 (-0.046288, 0.012548)	-	-	-	-
Houses in poor condition (%)	0.000307 (-0.038585, 0.010560)	-	-	-	-
Households renting (%)	0.000361 (-0.044591, 0.013485)	-	-	-	-
College education (%)	0.000062 (-0.044700, 0.014076)	-	-	-	-
Individuals below poverty level (%)	0.000664 (-0.048067, 0.011736)	-	-	-	-
Uninsured (%)	0.000202 (-0.045289, 0.009745)	-	-	-	-
Vacant houses (%)	0.000340 (-0.048853, 0.012497)	-	-	-	-
Walk score	0.000588 (-0.047642, 0.012196)	-	-	-	-
Households below poverty level (%)	0.000096 (-0.040107, 0.012468)	-	-	-	-
Poverty index	0.000373 (-0.044388, 0.012163)	-	-	-	-
Number supermarkets	0.000685 (-0.047478, 0.012122)	-	-	-	-

©2020 Krauland MG et al. *JAMA Network Open*

eTable 3. Continued

Determinant	Univariate	Income Model	Income and Education Model	Biological Model	Combined Social and Biological Model
Number fast food restaurants	0.000617 (-0.047383, 0.012217)	-	-	-	-
Households with no vehicle (%)	0.000132 (-0.040338, 0.013020)	-	-	-	-
Homicide counts	0.000526 (-0.047399, 0.010806)	-	-	-	-
Median age	0.000577 (-0.046617, 0.012231)	-	-	-	-
Obesity Rate (%)	0.000389 (-0.046331, 0.015452)	-	-	-	-

©2020 Krauland MG et al. *JAMA Network Open*

eTable 4. Correlation of Social Determinants by Pearson's Correlation Coefficient

	HS	C	FF	FS	PL	H	MA	MI	NV	Ob	PC	PI	PM	R	Sm	Ue	PPL	Ui	VH
C	0.693																		
FF	0.203	0.192																	
FS	-0.758	-0.656	-0.213																
PL	-0.710	-0.452	-0.130	0.843															
H	-0.419	-0.329	-0.022	0.571	0.530														
MA	0.228	-0.066	-0.012	-0.290	-0.490	-0.140													
MI	0.689	0.721	0.144	-0.714	-0.716	-0.398	0.287												
NV	-0.637	-0.338	-0.099	0.735	0.816	0.424	-0.452	-0.605											
Ob	-0.647	-0.650	-0.075	0.784	0.643	0.517	-0.194	-0.594	0.640										
PC	-0.539	-0.498	-0.188	0.617	0.611	0.332	-0.111	-0.496	0.583	0.540									
PI	-0.665	-0.735	-0.133	0.731	0.636	0.427	-0.142	-0.744	0.519	0.619	0.533								
PM	-0.266	-0.171	-0.112	0.333	0.342	0.233	-0.270	-0.370	0.313	0.207	0.214	0.219							
R	-0.520	-0.212	0.018	0.612	0.773	0.372	-0.642	-0.680	0.758	0.457	0.481	0.524	0.365						
Sm	0.153	0.153	0.422	-0.181	-0.151	-0.021	0.066	0.129	-0.149	-0.153	-0.073	-0.110	-0.091	-0.054					
Ue	-0.639	-0.561	-0.162	0.808	0.717	0.501	-0.245	-0.585	0.638	0.736	0.463	0.594	0.222	0.481	-0.150				
PPL	-0.205	-0.259	-0.164	0.270	0.126	0.132	0.049	-0.162	0.095	0.262	0.087	0.254	0.127	0.069	-0.043	0.434			
Ui	-0.567	-0.587	-0.152	0.557	0.477	0.344	-0.240	-0.664	0.441	0.473	0.465	0.601	0.251	0.507	-0.138	0.464	0.149		
VH	-0.522	-0.537	-0.231	0.677	0.555	0.493	-0.230	-0.593	0.447	0.508	0.578	0.616	0.284	0.450	-0.189	0.565	0.207	0.567	
WS	-0.164	0.101	0.059	0.225	0.371	0.103	-0.517	-0.373	0.453	0.104	0.112	0.139	0.367	0.667	-0.039	0.105	-0.081	0.301	0.213

KEY: HS, % High School Graduates; C, % College Graduate or Higher; FF, Number of fast food restaurants; FS, % Households receiving foodstamps; PL, % Households below poverty level; H, Homicides; MA, Median age; MI, Median income; NV, % Households with no vehicle; Ob, % Obese; PC, % Housing in poor condition ; PI, Poverty Index; PM, Particulate matter (Ppm2.5); R, % Renters; Sm, Number of supermarkets; Ue, % Unemployed; PPL, % People below poverty level; Ui, % Uninsured; VH, % Vacant housing; WS, Walk Score

©2020 Krauland MG et al. *JAMA Network Open*

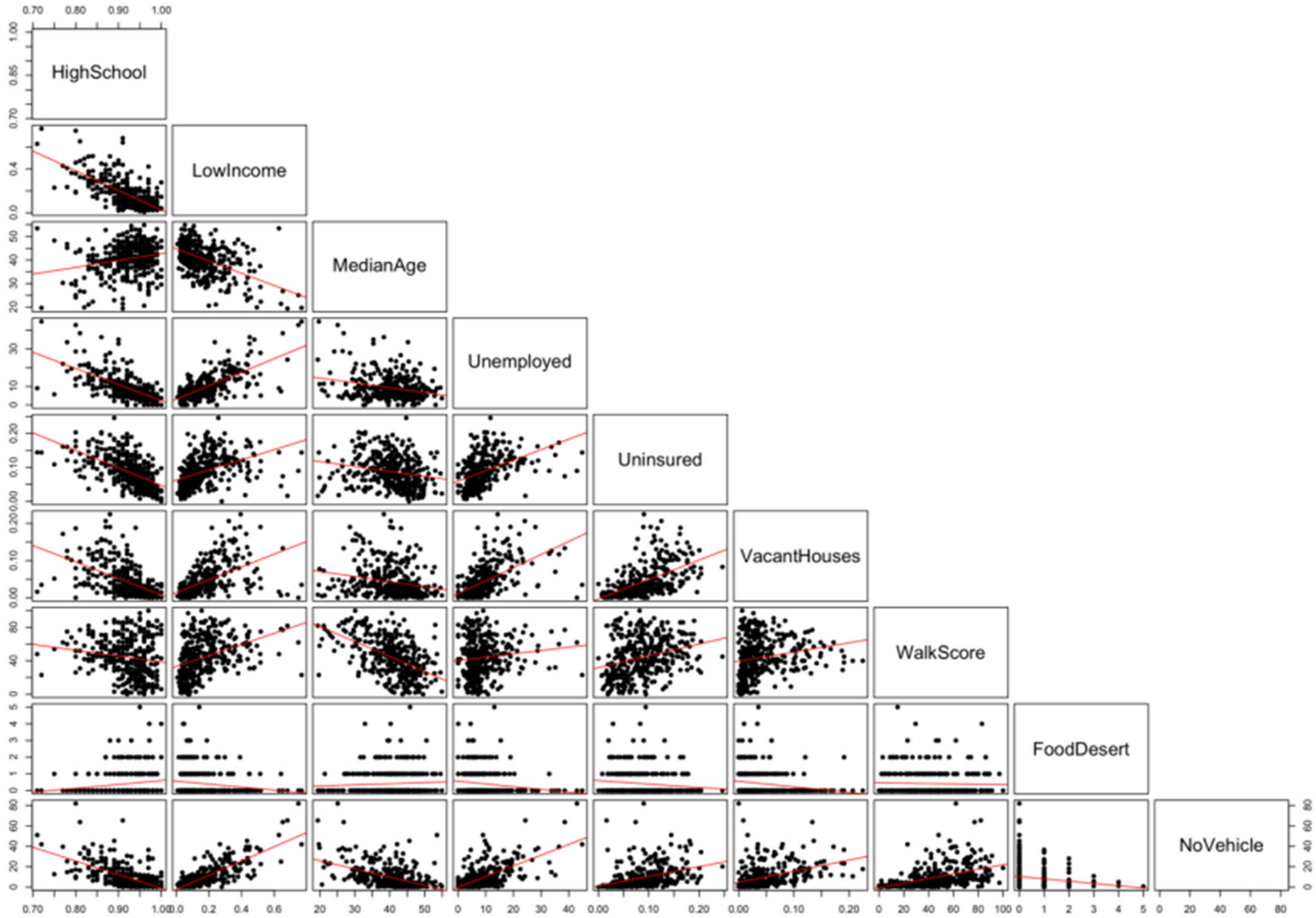

eFigure 6. Pairwise Plots of Selected Representative Social Determinants by Census Tract

Determinants include (in order from top to bottom): percent high school graduates; percent households below poverty level (LowIncome); median age; percent unemployed; percent uninsured; percent vacant housing, walk score; food desert (based on number of supermarkets); percent households with no access to vehicle. Regression line in red.

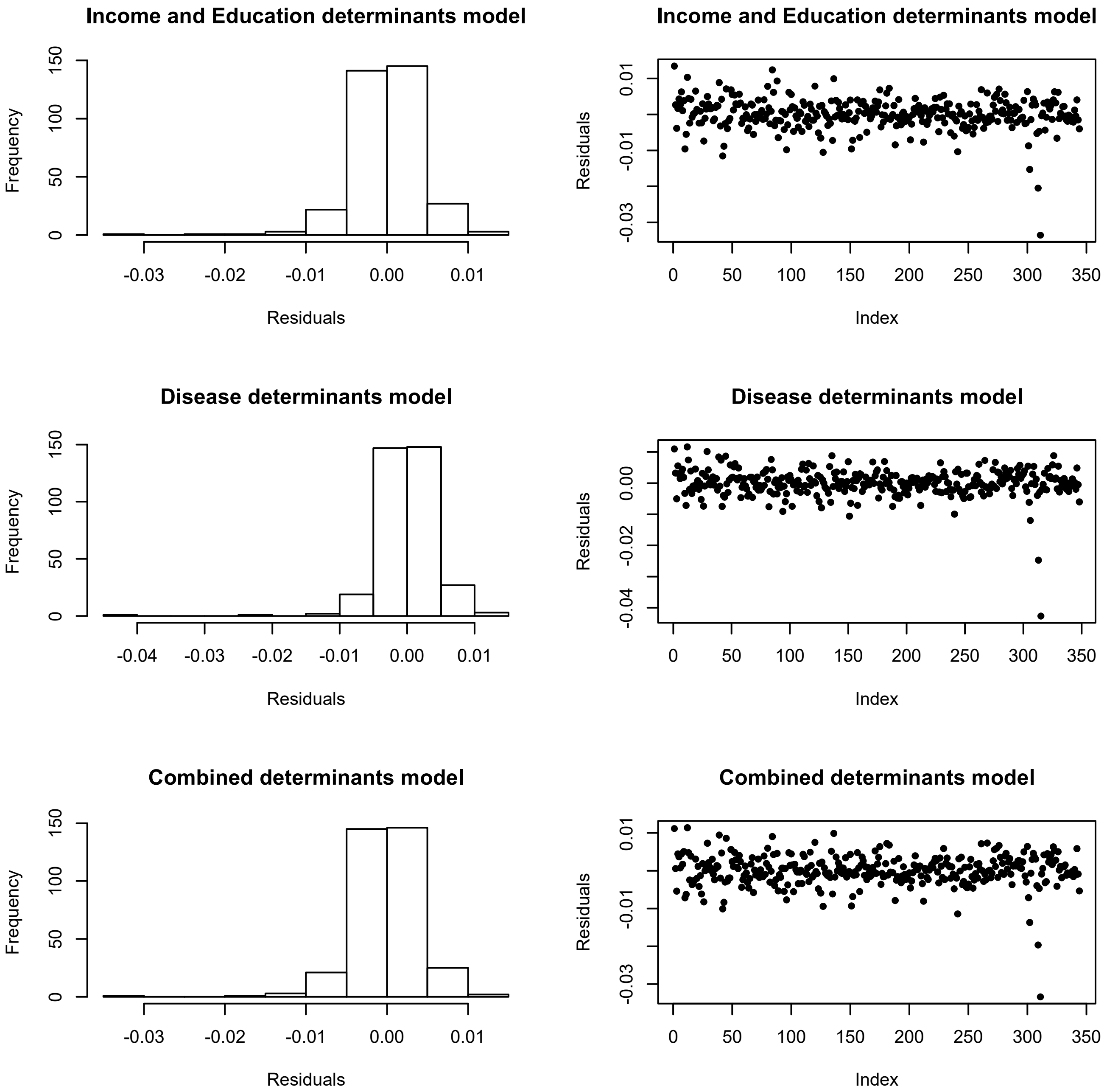

eFigure 7. Residual Analysis for Fit of Linear Models of Difference Between Expected and Observed Four-Year Cardiovascular Disease Death Risk by Social and Disease Determinants

©2020 Krauland MG et al. *JAMA Network Open*

eReferences

1. Grefenstette JJ, Brown ST, Rosenfeld R, et al. FRED (a Framework for Reconstructing Epidemic Dynamics): an open-source software system for modeling infectious diseases and control strategies using census-based populations. *BMC Public Health.* 2013;13:940.
2. Wheaton WD, Cajka JC, Chasteen BM, et al. Synthesized Population Databases: A US Geospatial Database for Agent-Based Models. *Methods Rep RTI Press.* 2009;2009(10):905.
3. National Health and Nutrition Examination Survey Data. U.S. Department of Health and Human Services, Centers for Disease Control and Prevention. https://wwwn.cdc.gov/nchs/nhanes/search/datapage.aspx?Component=Laboratory&CycleBeginYear=2009. Published 2010. Accessed.
4. National Health Interview Survey-2010. CDC - National Center for Health Statistics National Health Interview Survey Web site. https://www.cdc.gov/nchs/nhis/nhis_2010_data_release.htm. Published 2010. Accessed.
5. Pocock SJ, McCormack V, Gueyffier F, Boutitie F, Fagard RH, Boissel JP. A score for predicting risk of death from cardiovascular disease in adults with raised blood pressure, based on individual patient data from randomised controlled trials. *BMJ.* 2001;323(7304):75-81.
6. Piñeiro GP, S.; Guerschman,J.P.; Parueloa, J.M. How to evaluate models: Observed vs. predicted or predicted vs. observed? *Ecological Modelling.* 2008;216(3-4):316-322.
7. Ortega Hinojosa AM, Davies MM, Jarjour S, et al. Developing small-area predictions for smoking and obesity prevalence in the United States for use in Environmental Public Health Tracking. *Environ Res.* 2014;134:435-452.
8. Anselin L, Syabri I, Kho Y. GeoDa: An introduction to spatial data analysis. *Geogr Anal.* 2006;38(1):5-22.
